# Outcome analysis of breast cancer patients who declined evidence-based treatment

**DOI:** 10.1186/1477-7819-10-118

**Published:** 2012-06-26

**Authors:** Kurian Joseph, Sebastian Vrouwe, Anmmd Kamruzzaman, Ali Balbaid, David Fenton, Richard Berendt, Edward Yu, Patricia Tai

**Affiliations:** 1 Divisions of Radiation Oncology, University of Alberta, Edmonton, AB, Canada; 2 Medical Oncology, University of Alberta, Edmonton, AB, Canada; 3 Laboratory Medicine & Pathology, Cross Cancer Institute, University of Alberta, Edmonton, AB, Canada; 4 Laboratory Medicine & Department of Public Health Sciences, University of Alberta, Edmonton, AB, Canada; 5 Department of Radiation Oncology, London Regional Cancer Center, London, ON, Canada; 6 Department of Radiation Oncology, Allan Blair Cancer Center, Regina, Canada

## Abstract

**Background:**

To analyze the characteristics and outcomes of women with breast cancer in the Northern Alberta Health Region (NAHR) who declined recommended primary standard treatments.

**Methods:**

A chart review was performed of breast cancer patients who refused recommended treatments during the period 1980 to 2006. A matched pair analysis was performed to compare the survival data between those who refused or received standard treatments.

**Results:**

A total of 185 (1.2%) patients refused standard treatment. Eighty-seven (47%) were below the age of 75 at diagnosis. The majority of those who refused standard treatments were married (50.6%), 50 years or older (60.9%), and from the urban area (65.5%). The 5-year overall survival rates were 43.2% (95% CI: 32.0 to 54.4%) for those who refused standard treatments and 81.9% (95% CI: 76.9 to 86.9%) for those who received them. The corresponding values for the disease-specific survival were 46.2% (95% CI: 34.9 to 57.6%) vs. 84.7% (95% CI: 80.0 to 89.4%).

**Conclusions:**

Women who declined primary standard treatment had significantly worse survival than those who received standard treatments. There is no evidence to support using Complementary and Alternative Medicine (CAM) as primary cancer treatment.

## Background

Breast cancer is the second leading cause of cancer-related death (14%) among Canadian women, with an estimated 23,400 new cases and 5,100 deaths in 2011.^1^ In Canada, 1 in 9 (11%) women is expected to develop breast cancer during their lifetime, and 1 in 29 (4%) die of it [1]. Breast cancer management involves either modified radical mastectomy (MRM) or breast conservation surgery (BCS) as the primary treatment modality followed by adjuvant treatments based on pathological characteristics. A 20-year update of the National Surgical Adjuvant Breast and Bowel Project (NSABP) study reported an overall survival benefit of 45–49% among women treated with mastectomy, and 44–48% survival benefit for those underwent lumpectomy and breast irradiation [2]. In case of locally advanced or inoperable breast cancer, neoadjuvant chemotherapy or radiotherapy has been considered for downsizing and downstaging the tumor. Kuerer et al. reported an overall 5-year survival rate of 82% and 66%, respectively, when segmental mastectomy with axillary dissection or modified radical mastectomy was used, following neoadjuvant chemotherapy [3].

Despite all efforts of the physicians, some patients may decline the offered standard treatments. Although a patient’s decision to refuse cancer treatment may be hard to accept by the physician, this option is well within their rights. Women with a diagnosis of breast cancer can refuse their treatment partly or completely. The reasons for such decisions could be multifactorial. Little attention is devoted to understanding why and when cancer patients refuse their offered treatment modalities. Information in the literature is scanty too. This population-based study analyzes the characteristics and outcomes of women with breast cancer in the Northern Alberta Health Region (NAHR) who refused recommended primary standard treatments. Insights gained would be extremely useful in the clinic.

## Methods

### Data

This is a retrospective chart review of patients diagnosed with breast cancer in the NAHR, during the period 1980 to 2006, who refused the standard treatment recommendations and were retrieved from the population-based Alberta Cancer Registry (ACR) following approval from the research ethics board. Any patient who has completely refused the recommended standard primary treatment plan following biopsy confirmation of breast cancer is considered as refusal of standard treatment. Primary treatment could be surgery, neoadjuvant radiotherapy or chemotherapy. Patients who refused adjuvant treatments following surgery were not included in this analysis. We also excluded patients older than 75 years in our study as they have generally been eliminated from clinical studies and active treatment regimens (e.g., chemotherapy) in the past. The following data were compiled by chart review: patient’s demographics at diagnosis, tumor characteristics, initial clinical and pathologic stages, available details of any other treatment received, all recurrence/relapse details and treatments received, date of last contact or follow-up, and disease status if alive or date and cause of death.

### Statistical analysis

Demographics and characteristics of the study patients are reported. A matched analysis was performed to compare the outcome between patients who refused the recommended treatment and those who received standard primary treatment during the study period. To maximize the statistical power, the matching ratio was set to 5:1; i.e., against each refusal five non-refusals are selected. The matching variables were: age (± 3 years), calendar year and clinical stage at diagnosis. Specific tumor node metastasis (TNM) variables were not used for matching since  the majority of the patients had biopsy confirmation only. A *p*-value ≤ 0.05 was considered statistically significant.

We compared the survival between groups using the Kaplan-Meier survival method. All the analyses were completed in SAS software version 9.1 (SAS Institute, NC, USA).

## Results

The demographics of the patients who refused standard surgical treatments in the NAHR during the period 1980–2006 are listed in Table 1. A total of 185 (1.2%) patients refused the recommended standard primary treatment in the NAHR. Of them, 87 (47%) were below the age of 75 at the time of diagnosis.

**Table 1:** Demographics and characteristics of women diagnosed with breast carcinoma in the NAHR and refused primary standard treatment, 1980–2006

Total number of women diagnosed with breast carcinoma	15,427
Women who refused primary standard treatment	185 (1.2%)
Women of age <75 years at diagnosis among those who refused primary standard treatment	87 (47.0%)
Characteristics of women (age <75 years at diagnosis) who refused primary standard treatment	*n* =87 (%)
Age at diagnosis	
Less than 50 years	34 (39.1)
50 to 75 years	53 (60.9)
Treatment	
CAM	50 (57.5)
Unknown	37 (42.5)
Marital status	
Single	14 (16.1)
Married	44 (50.6)
Separated	22 (25.3)
Other	7 (8.1)
Occupation	
Home maker	33 (37.9)
Retired	9 (10.4)
Other	34 (39.1)
Unknown	11 (12.7)
Residence at diagnosis	
Urban	57 (65.5)
Rural	30 (34.5)
Vital status	
Alive	21 (24.1)
Deceased	66 (75.9)
Causes of death (*n =* 66)	
Metastatic breast cancer	61 (70.1)
Other	5 (5.8)

*NAHR*: Northern Alberta Health Region.

*CAM*: Complementary & alternative medicine.

The majority of the women who refused standard treatments were married (50.6%), 50 years and older (60.9%), and urban residents (65.5%) at the time of diagnosis. Among the retired group of patients, 10.4% refused the standard treatment.

Table 2 describes the stage of disease at initial presentation and disease status on referral to the tertiary cancer center. Fifty-seven patients (65.5%) had biopsy confirmation only; 30 (34.5%) underwent delayed surgery. Fifty patients (58%) decided to undergo complementary and alternative medicine (CAM) treatments, whereas the reason for refusal was unclear for 37 (42.5%) patients. There was on average a delay of 20–30 weeks before surgery.

**Table 2:** Stage of disease at initial presentation and status of disease on referral to the tertiary cancer center, among women diagnosed with breast carcinoma (age <75 years at diagnosis) who refused primary standard treatments in the NAHR, 1980–2006

**Clinical stage of patient group at initial presentation**	**Number of patients by stage of disease**		
	**Number of patients** (***n*** **=** **87**)	**Duration of CAM therapy or delaying treatment (in weeks) Median (range)**	**Stage on referral to the cancer clinic**
Biopsy only (*n =* 57)			
I (including DCIS)	16	62 (41–101)	II/III A/B (6)
II	22	Unknown	IV
III	19	Unknown	IV
IV	0	Unknown	-

*Unknown* patients do not return for follow-up evaluation; *CAM* complementary and alternative medicine; *RT* radiotherapy; *CT* chemotherapy range (= difference between maximum and minimum values).

**Figure 1:**
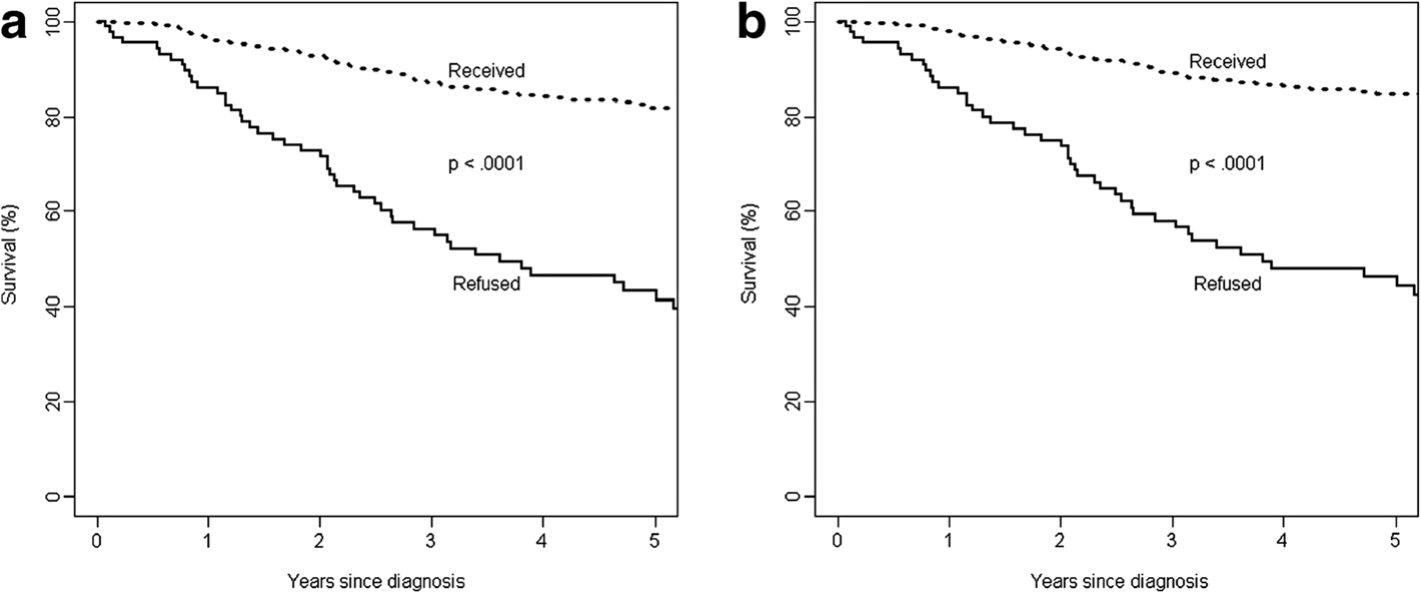
Survival of the breast cancer patients who received standard treatment compared to those who refused treatment in NAHR, 1980–2006. (a) All causes of deaths and (b) deaths due to breast cancer only.

Figure 1 compares the survival curves of patients who refused and accepted treatments. The 5-year overall survival was 43.2% (95% CI: 32.0 to 54.4%) for those who refused standard treatments and 81.9% (95% CI: 76.9 to 86.9%) for those who received them. The corresponding values for the disease-specific survival were 46.2% (95% CI: 34.9 to 57.6%) vs. 84.7% (95% CI: 80.0 to 89.4%).

**Figure 2:**
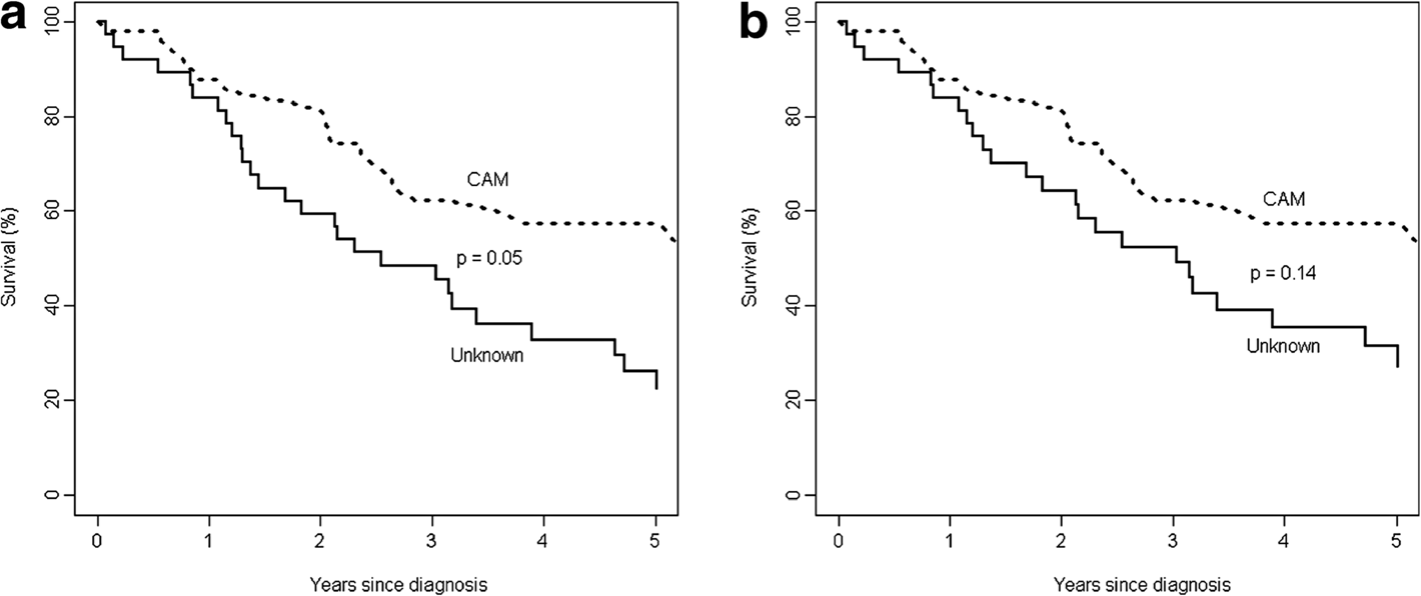
Survival of the breast cancer patients who took CAM compared to those who did not take CAM in NAHR, 1980–2006; (a) all causes of deaths and (b) deaths due to breast cancer only.

Since 58% of patients received different kinds of CAM, a comparison of the outcome was performed between groups who received CAM and those whose treatment details were not known. Figure 2 compares the survival patterns of women who refused treatment who either received CAM or for whom the reason for refusal was unknown. The 5-year overall survival was 57.4% (95% CI: 42.7 to 72.1%) for women who received CAM and 26.3% (95% CI: 11.3 to 41.3%) for those whose treatment details were unknown. The global survival for the CAM group was better than for women whose reason for refusal was unknown (*p* ≤ 0.05), and disease-specific survival for the CAM was better for women whose reason for refusal was unknown, but this was not statistically significant (31.5%; 95% CI: 15.1 to 48.0%).

## Discussion

Our study was designed to quantify the incidence and characteristics of breast cancer patients who refused standard treatments offered in the NAHR and to compare their outcomes with those who received the recommended treatments. At present, a reasonable estimate of the incidence of patients refusing standard breast cancer treatments is not available. Verkooijen et al. reported that 0.7% of the breast cancer patients registered at the Geneva cancer registry had declined any of the standard treatments offered [4,5]. The disease-specific survival was significantly lower for this group of patients when compared to those who received standard treatment (36% vs. 75% at 10 years). Our data showed that 1.2% breast cancer patients refused standard treatments with similarly poor outcomes (43.2% vs. 81.9%).

A patient’s decision to decline the recommended cancer treatment depends on many factors, probably based on previous experiences and personal values [6]. Various factors claimed to be associated with cancer treatment refusal include: lower social class, higher education, single or divorced, patients living in a rural community, higher age group, medical co-morbidity, fear of surgery, fear of anesthesia and fear of treatment-related side effects [4-7]. No significant association was linked to our population, and fewer incidences occurred among the retired group. The majority of our cohort was 50 years or older, lived in the urban community and was married. Our study did not reveal any factors closely related to cancer treatment refusal.

Growing numbers of patients globally are integrating non-standard treatments such as herbal medicines, vitamins, special diets, prayers, etc., into their cancer care. These non-standard treatments are considered complementary and alternative medicine (CAM). One of the interesting observations was that the majority of our patients who refused the standard treatments received various types of CAM. Unfortunately, the details of CAM received by our patients were not available, since most of these patients refused to be followed up. In our series, most of the patients failed to report back for follow-up once they decided to refuse surgery. There is widespread concern among oncologists regarding the use of CAM for cancer treatment as primary or supportive medication because of the lack of quality data supporting its clinical efficiency. Patients may be aware of the concerns felt by their oncologist regarding the use of CAM, and hence such patients are often reluctant to disclose information on the type of therapies they seek. Kremser et al. reported that a significant proportion (87.5%) of women with breast cancer use complementary therapies as supportive treatment [8]. Although some benefits of CAM as a supportive treatment have been reported, most evidence demonstrates that CAM is ineffective or could adversely interact with standard cancer treatment [8].

Our data showed that almost all the patients who initially refused treatment progressed to a higher stage on later presentation at the cancer center. The majority of the patients (57%) in our series initially chose CAM as the primary treatment instead of surgery. Those who had chosen CAM had disease progression with particularly poor disease-specific survival when compared to those who received standard treatment. It is therefore important to educate patients who decided to decline standard treatments for CAM about the risk of such therapies. There could be many reasons for a patient to decline the standard treatment offered. However, a decision to refuse a possible curative treatment could lead to an ethical dilemma for the physician. In general, the oncologist may respect a patient’s decision and disengage with them. However, this may not be in the best interest of the patient. Carrese et al. recommended evaluating the patient’s capacity for decision-making and exploring the potential risk factors influencing the decision to refuse treatment [9]. Most patients appreciate effective and open communication by the oncologist, which is essential for better decisions and outcomes. Family physicians (FPs) can also play significant roles in the decision-making concerning their patient’s cancer treatment, since most of them have long, trusting relationships with their patients. In general, oncologists provide detailed information to the FPs about their patient’s treatment plan after the initial consultation. FPs can provide emotional support and educate the patient about the disease and the nature of treatment needed.

This study was discussed at the Canadian Radiation Oncologist’s (CARO) 2011 annual meeting, especially the role of the involved oncologist in managing patients who refuse cancer treatment. In general, the forum agreed on the need for carefully educating the patient directly and through the FP about the risks of taking CAM instead of evidence-based treatments as primary treatment. Such discussions could lead to reversal of patient’s decision to refuse treatment. Open communication between the physician and patient is important. Poor communication was mentioned as a reason for declining conventional treatment [10]. Psychosocial services can also help to bridge the communication gap and resolve any misunderstanding of treatment options. In our study, surgery was delayed an average of 20–30 weeks because of initial treatment with CAM. Patients could be interested in experiencing the benefits of trying CAM before surgery because of the belief that CAM could result in a cure. The physician should emphasize the impact of delaying radical treatment, with the risk of forfeiting the possibility of conservative surgery, or increasing nodal or distant cancer spread.

A potential limitation of our study was that it was a retrospective series with a relatively small sample size. In addition, the study lacked co-morbidity information as well as detailed information on non-standard treatments received by each patient.

## Conclusions

In summary, women who refused evidence-based treatment for breast cancer had significantly worse outcomes than women who received standard therapies. At present, there is no evidence available to support the use of CAM as the primary breast cancer treatment. It is therefore important for the physician to educate patients about the treatments and also to warn about the potential risk of using CAM instead of undergoing evidence-based treatment.

## Competing interest

The authors indicated no potential conflicts of interest.

## Authors’ contributions

**Conception and design:** KJ, SV, PT. **Administrative support:** KJ, SV, AK, AB. **Provision of study materials or patients:** KJ, SV, AK. **Collection and assembly of data:** SV, AK. **Data analysis and interpretation:** KJ, AK, PT. **Manuscript writing:** KJ, SV, AK. **Review of manuscript:** DF, PT, RB, EY. **Final approval of manuscript:** KJ, SV, AK, AB, DF, PT, RB, EY. All authors read and approved the final manuscript.

This project was supported by the Health Quality Council of Alberta (HQCA). Partly presented at the annual CARO meeting 2011, Winnipeg, Manitoba, Canada.

## References

[B1] Canadian cancer statistics 2011 2011 Toronto, ON, Canada

[B2] Fisher B Anderson S Bryant J Twenty-year follow-up of a randomized trial comparing total mastectomy, lumpectomy, and lumpectomy plus irradiation for the treatment of invasive breast cancer N Engl J Med 2002 347 1233 1241 10.1056/NEJMoa022152 12393820

[B3] Kuerer HM Newman LA Smith TL Clinical course of breast cancer patients with complete pathologic primary tumor and axillary lymph node response to doxorubicin-based neoadjuvant chemotherapy J Clin Oncol 1999 17 2 460 469 10080586 10.1200/JCO.1999.17.2.460

[B4] Chang EY Glissmeyer M Tonnes S Outcomes of breast cancer in patients who use alternative therapies as primary treatment Am J Surg 2006 192 471 473 10.1016/j.amjsurg.2006.05.013 16978951

[B5] Verkooijen HM Fioretta GM Rapiti E Patients’ refusal of surgery strongly impairs breast cancer survival Ann Surg 2005 242 276 280 10.1097/01.sla.0000171305.31703.84 16041219 PMC1357734

[B6] van Kleffens T van Leeuwen E Physicians’ evaluations of patients' decisions to refuse oncological treatment J Med Ethics 2005 31 131 136 10.1136/jme.2004.008755 15738431 PMC1734113

[B7] Huchcroft SA Snodgrass T Cancer patients who refuse treatment Cancer Causes and Control 1993 4 179 292 8318634 10.1007/BF00051311

[B8] Kremser T Evans A Moore A Use of complementary therapies by Australian women with breast cancer Breast 2008 17 387 394 10.1016/j.breast.2007.12.006 18534852

[B9] Carrese JA Refusal of care: patients' well-being and physicians' ethical obligations: "but doctor, I want to go home" JAMA 2006 296 6 691 695 10.1001/jama.296.6.691 16896112

[B10] Shumay DM Maskarinec G Kakai H Gotay CC Why some cancer patients choose complementary and alternative medicine instead of conventional treatment J Fam Pract 2001 50 1067 11742609

